# Genome-Wide Estimation of the Spontaneous Mutation Rate of Human Adenovirus 5 by High-Fidelity Deep Sequencing

**DOI:** 10.1371/journal.ppat.1006013

**Published:** 2016-11-08

**Authors:** Jennifer Risso-Ballester, José M. Cuevas, Rafael Sanjuán

**Affiliations:** 1 Institute for Integrative Systems Biology (I2SysBio), Universitat de València and Consejo Superior de Investigaciones Científicas, València, Spain; 2 Departament de Genètica, Universitat de València, València, Spain; Rutgers University, UNITED STATES

## Abstract

Rates of spontaneous mutation determine the ability of viruses to evolve, infect new hosts, evade immunity and undergo drug resistance. Contrarily to RNA viruses, few mutation rate estimates have been obtained for DNA viruses, because their high replication fidelity implies that new mutations typically fall below the detection limits of Sanger and standard next-generation sequencing. Here, we have used a recently developed high-fidelity deep sequencing technique (Duplex Sequencing) to score spontaneous mutations in human adenovirus 5 under conditions of minimal selection. Based on >200 single-base spontaneous mutations detected throughout the entire viral genome, we infer an average mutation rate of 1.3 × 10^−7^ per base per cell infection cycle. This value is similar to those of other, large double-stranded DNA viruses, but an order of magnitude lower than those of single-stranded DNA viruses, consistent with the possible action of post-replicative repair. Although the mutation rate did not vary strongly along the adenovirus genome, we found several sources of mutation rate heterogeneity. First, two regions mapping to transcription units L3 and E1B-IVa2 were significantly depleted for mutations. Second, several point insertions/deletions located within low-complexity sequence contexts appeared recurrently, suggesting mutational hotspots. Third, mutation probability increased at GpC dinucleotides. Our findings suggest that host factors may influence the distribution of spontaneous mutations in human adenoviruses and potentially other nuclear DNA viruses.

## Author Summary

Next-generation sequencing has provided a powerful tool for studying microbial genetic diversity but suffers from relatively low per-base accuracy, limiting our ability to detect low-frequency polymorphisms and spontaneous mutations. However, this limitation has been solved recently by the development of high-fidelity deep sequencing techniques. Taking advantage of these advancements, here we provide the first unbiased genome-wide characterization of the rate of spontaneous mutation of a human DNA virus (adenovirus 5) under controlled laboratory conditions. The adenovirus genome shows a relatively low mutation rate, consistent with high replication fidelity and the action of post-replicative repair. We also found evidence for mutation rate heterogeneities and regions of genetic instability in the viral genome. Together with previous reports, our findings indicate that DNA viruses with large double-stranded genomes mutate significantly slower than those with small single-stranded genomes.

## Introduction

DNA viruses have been traditionally viewed as slowly-evolving entities, but this notion has been challenged in the last decade after the discovery of several highly diverse and fast-evolving DNA viruses [1–6]. The pace of evolution should be dependent on the rate at which new spontaneous mutations are produced, yet it is currently accepted that DNA virus mutation rates are typically much lower than those of RNA viruses [7]. However, as opposed to RNA viruses, few mutation rate estimates have been obtained for DNA viruses, which include four bacteriophages (φX174, m13, λ, and T4), herpes simplex virus, and human cytomegalovirus [7–11]. Moreover, these estimates were derived from indirect, phenotype-based methods of mutation detection, used very small portions of the viral genome, or suffered from bias due to selection acting on population mutation frequencies. Therefore, we currently lack an unbiased, genome-wide view of how spontaneous mutations are produced in DNA viruses. Although next-generation sequencing (NGS) has made it possible to analyze genetic variation in full-length DNA virus genomes with unprecedented detail, its relatively low per-read accuracy has prevented detection of rare variants, including new spontaneous mutations. This problem has been solved in recently-developed methods that increase the accuracy of NGS by orders of magnitude [12,13], now permitting an in-depth characterization of DNA virus spontaneous mutation rates.

Adenoviruses are non-enveloped icosahedral, viruses with double-stranded linear DNA genomes of 26–45 kbp. They infect a broad range of vertebrates, and over 60 serotypes of human adenoviruses have been identified and grouped into seven species (A-G) and serotypes [14]. Human adenoviruses can cause a wide variety of diseases including eye, gut and respiratory infections that are typically not clinically relevant for healthy individuals, yet potentially life-threating for immuno-compromised patients [15]. The adenovirus genome is flanked by inverted terminal repeats (ITR) containing the origins of replication and encodes early-expressed proteins required for DNA replication (transcription units E1A, E1B, E2, E3 and E4), late structural proteins (transcription units L1, L2, L3, L4 and L5), and intermediate-expressed proteins (IX and IVa2). Transcription units can be oriented in either direction and undergo extensive alternative splicing to yield diverse mRNA products, referred to as genes. Adenoviruses constitute an excellent model for studying the evolution of DNA viruses due to their high prevalence, broad tropism, tractability, and relatively simple genomes. Sequencing of isolates from patients has revealed peaks of genetic diversity at some regions of the hexon, fiber, penton base genes, and E3, which encode exposed domains of the capsid required for host cell binding and entry, or proteins involved in virus-host interactions and immune evasion [16,17]. Some of these hypervariable regions are being currently used for genotyping adenovirus isolates by PCR and Sanger sequencing, as well as by NGS [18].

Here, we have used high-fidelity NGS to analyze the genome-wide rate of spontaneous mutation of human adenovirus C5 (HAdv5) under controlled laboratory conditions. After an endpoint dilution step to remove pre-existing diversity and short-term culturing to minimize the effects of natural selection, we sequenced >98% of the viral genome with >1000-fold coverage and extremely high accuracy. This allowed us to identify >200 spontaneous mutations produced at different positions of the viral genome. The estimated rate of spontaneous mutation was 1.3 × 10^−7^ per site per cell infection cycle, and the mutational spectrum was dominated by G-to-A and C-to-T base transitions. We found that different transcriptions units mutated at roughly similar rates, indicating that hypervariable regions originate mainly by selection and not by mutational hotspots. However, we found several sources of mutation rate heterogeneity. First, GpC dinucleotides showed increased mutation probability. Second, we identified two 5 kpb regions approximately mapping to E1B, IX, IVa2 and L3 which showed a significant reduction in the number of accumulated mutations. Third, several individual genome sites located within low-complexity sequence contexts exhibited the same mutations recurrently in independently replicating viruses, suggesting the presence of mutational hotspots. Some of these mutation rate heterogeneities correlated with changes in diversity in publicly available patient-derived sequences.

## Results

### Detection of HAdv5 mutations by high-fidelity deep sequencing

**Fig 1:**
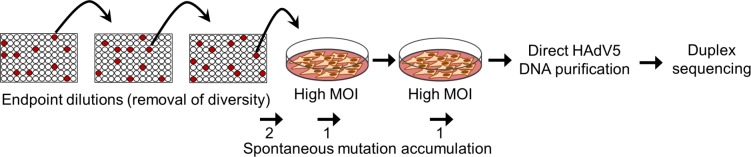
Experimental setup for HAdv5 mutation rate measurement. Three serial endpoint dilution transfers were carried out in 96-well plates (hypothetical infected wells are depicted in red) and then passaged twice in 10-cm plates at high MOI. Each endpoint dilution should remove pre-existing diversity. The estimated number of infection cycles during which mutations could accumulate are shown below: two for growth of the virus from a single PFU in the last endpoint dilution step, and one for each high-MOI transfer. Viral DNA was purified and used directly for Duplex Sequencing. The entire experiment (endpoint dilutions, amplification, and sequencing) was repeated three times (R1, R2, and R3).

HAdv5 was subjected to three serial end-point dilution steps in HeLa cells and then re-amplified by two serial transfers in liquid culture at high multiplicity of infection (MOI) to obtain sufficient viral genome copies to carry out DNA extraction and NGS without PCR amplification, such that we could avoid PCR-driven sequencing errors (**Fig 1**).

**Fig 2:**
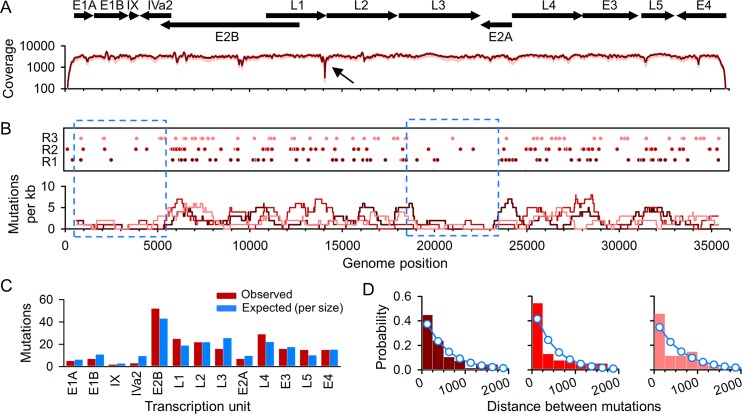
Distribution of spontaneous mutations along the HAdv5 genome. **A.** Genetic map and sequencing coverage. The protein-coding regions of different transcription units are indicated on top with arrows showing their orientation. The dark red line indicates the median coverage from three biological replicates, and the light red line indicates the lowest and highest coverage values. A sharp coverage drop was found around genome position 14,073 (arrow). **B.** Mutations observed along the viral genome for each of three independent biological replicates (R1, R2, R3). Each circle represents a nucleotide substitution. Insertions/deletions are not plotted. The substitution count averaged over a 1 kpb sliding window (dark red: R1; bright red: R2, light red: R3) is shown below. The dotted blue squares indicate two regions of low mutation accumulation. **C.** Observed mutation counts per transcription unit (coding regions) versus expected counts assuming a constant genome-wide mutation rate. **D.** Distribution of the distance between consecutive mutations (color codes are as in **B**). Histograms show the observed distances, and the superimposed blue dots/lines correspond to a geometric distribution (null model).

The endpoint dilution steps ensured that viral growth was initiated from a single infectious unit. Since, after three serial endpoint dilutions, all pre-existing genetic diversity should have been removed, variants observed in the sequenced populations should correspond to newly-produced, spontaneous mutations. HAdv5 DNA was purified from the cytoplasm of infected cells to avoid carrying over large amounts of nuclear cellular DNA, and directly subjected to Duplex Sequencing (DS) using the Illumina platform. DS relies on template tagging and strand complementarity to increase base call accuracy by orders of magnitude compared to conventional NGS [13,19]. For each of three independent biological replicates, 99% of the HAdv5 genome was sequenced with an average coverage >2500 in each replicate (**Fig 2A)**. We found 68, 78, and 62 different single-base substitutions in 93.2, 115.7, and 123.7 Mb sequenced, respectively, yielding an average per-base mutation frequency of (6.4 ± 0.7) × 10^−7^ (**Table 1)**.

**Table 1:** Spontaneous mutational spectrum of HAdv5.

Mutation	Replicate assay			Total %
	1	2	3	
G→A	11	22	13	22.1
C→T	13	13	17	20.7
A→G	9	17	6	15.4
T→C	6	5	7	8.7
G→T	8	2	1	5.3
C→A	8	5	4	8.2
T→G	2	3	3	3.8
A→C	2	2	3	3.4
G→C	2	4	3	4.3
C→G	2	2	1	2.4
A→T	4	1	1	2.9
T→A	1	2	3	2.9
Total	68	78	62	100.0
Mbp sequenced	93.2	115.7	123.7	-
Mutation frequency (× 10^6^)	0.73	0.67	0.50	-

These mutations were present at frequencies below 1% in sequence reads (**S1 Table**). Hence, it is highly unlikely that these were pre-existing polymorphisms carried forward from the initial population, because any variant that may have survived the three endpoint dilutions should have reached very high population frequencies after such drastic bottlenecks. To test whether the observed variants were sequencing errors, we performed DS of a purified *E*. *coli* plasmid pUC18, which should exhibit low diversity given the high replication fidelity of bacteria. This yielded three single-nucleotide substitutions in 21.0 Mbp sequenced, which implies a maximal per-base sequencing error rate of 1.4 × 10^−7^ assuming that the plasmid contained no diversity. Thus, at least 80% of the observed variants should correspond to real mutations. Based on the estimated sequencing error, the net mutation frequency was (6.4–1.4) × 10^−7^ = 5.0 × 10^−7^. In addition to single-nucleotide substitutions, the HAdv5 sequences contained 70 point insertions and deletions (**S1 Table**), but these types of mutations were present in the control plasmid at similar frequencies, indicating that most should be sequencing errors. Therefore, we did not use insertions and deletions for mutation rate estimation. To further check the nucleotide substitutions detected by DS, we set out qPCR assays aimed at selectively amplifying wild-type and mutant alleles in four genome sites. For mutations detected by DS, the Ct values obtained in qPCRs designed to amplify the mutant allele were delayed by approximately 6 to 12 cycles compared to those in which the wild-type sequence was amplified. In contrast, for mutations not detected by DS, this difference increased to 12–20 cycles (**S1 Fig**). This further supports the conclusion that most DS mutations were real.

### HAdv5 spectrum and rate of spontaneous mutations

G→A transitions and the reverse complementary C→T transitions were the most abundant type of mutations detected by DS (42%), followed by A→G/T→C (24%), whereas transversions were 2.0 times less abundant than transitions (**Table 1**). We found that the nearly twofold excess of G→A and C→T changes over A→G/T→C was accounted for by a higher mutation probability at GpC motifs. Specifically, of the 3167 such dinucleotides in the HAdv5 genome, 50 contained G→A or C→T mutations, whereas the 13,380 G and C bases that were not part of GpC motifs showed only 39 mutations, revealing a 2.7-fold excess probability of G→A /C→T mutation at GpC dinucleotides (Fisher test: *P* < 0.001). In contrast, CpG motifs only showed a 1.3-fold excess C→T/G→A mutation probability compared to other G and C bases. The observed mutational pattern at GpC motifs is unlikely to be explained by selection, because HAdv5 was passaged only twice after the bottleneck, minimizing the ability of selection to purge spontaneous mutations. Furthermore, these passages were done at high MOI, which reduces the efficacy of selection [20–23]. The lack of significant selection was further supported by analysis of synonymous and non-synonymous variation. Based on the observed mutational spectrum, we expected 71.5% of base substitutions at coding regions to be non-synonymous under a neutral model. The observed fraction was 74.5%, a value that did not deviate significantly from this expectation (dN/dS = 1.16, chi-square test: *P* = 0.327). In the absence of selection, the observed mutation frequency should equal the mutation rate per cell infection cycle times the number of infection cycles elapsed [7]. Each of the two high-MOI transfers allowed for one infection cycle and, using our estimate of the HAdv5 burst size (ca. 10^4^ infectious units per cell), approximately two additional cycles were required for growth of the initial infectious unit isolated by endpoint dilution (see Methods). Hence, the calculated point mutation rate per cell infection cycle was 5.0 × 10^−7^ / 4 = 1.3 × 10^−7^.

### Distribution of spontaneous mutations along the HAdv5 genome

Genetic diversity is not uniformly distributed in adenovirus genomes [16,17], raising the question whether mutation rates vary accordingly. Although mutations were generally well-scattered along the HAdv5 genome, we found two regions of approximately 5 kpb showing significantly fewer mutations than expected under the assumption of a constant rate (**Fig 2B**). Specifically, the region encompassing genome sites 18,500 to 23,500 showed only seven total base substitutions in the three biological replicates, whereas the expected number was 29.17 (Binomial test: *P* < 0.001), the reduction being statistically significant in each of the three replicates (*P* ≤ 0.014). This region approximately maps to genes located in transcription unit L3, which encodes a protease and two capsid proteins (VI and hexon protein).

Another low-mutation region encompassed genome sites 500 to 5500, which includes transcription units E1B, IX, and IVa2 (Binomial test: *P* = 0.001), although in this case the reduction was significant in only two of the three replicates. One possible factor driving mutation depletion may be purifying selection acting specifically at these regions. However, the percentage of non-synonymous mutations in L3/E1B-IVa2 was similar to the rest of the genome (76.9% versus 74.5%, respectively), suggesting no differential selection pressure at the protein-coding level. We also found no major differences in the frequency of GpC dinucleotides between the low-mutation regions and the rest of the genome, and the reduction in mutation frequency in L3 was still significant after removing all G→A /C→T changes from the analysis (Binomial test: *P* < 0.001). Hence, the mechanisms driving mutation rate variation along the viral genome remain unclear. Aside from the reduced mutation frequency in these two regions, the observed number of mutations per transcription unit correlated well with the expected number assuming a constant mutation rate (**Fig 2C**; Chi-square test: *P* = 0.672). Finally, to further test for mutation clustering, we obtained the empirical distribution of the distance between consecutive mutations. Assuming a constant rate, this distance should follow a geometric distribution with parameter equal to the per-site mutation probability. The data were in broad agreement with this null model, although there was a slight increase in the proportion of mutations showing small distances, suggesting some level of clustering.

### Candidate mutational hotspots

Interestingly, we identified 11 individual genome sites that exhibited the exact same mutation in at least two of the three biological replicates, suggesting the presence of mutational hotspots (**Table 2**). For instance, genome site 9417, which maps to transcription unit E2B (terminal protein precursor gene) showed a T-to-G substitution in 0.28%, 0.54% and 0.77% of the reads of each replicate, a frequency that exceeds the genome-wide mutation frequency by orders of magnitude. This site was flanked by a G-rich motif (GGTGGGG), such that the mutation produced a G heptamer. Most other mutations were point insertions. A common feature of these recurrently appearing mutations was a low-complexity sequence context with frequent homopolymeric runs. This type of sequence context reduces replication fidelity by inducing frequent polymerase slippage, but also elevates the sequencing error rates. Based on this, we cannot rule out the possibility that these mutations were sequencing artefacts. In genome site 14,073, which maps to the end of L1, we found a frequent insertion that was further accompanied by a marked decrease in sequencing coverage, from >1000 to approximately 200 (**Fig 2A**).

**Table 2:** Mutations appearing recurrently in independent experiments and polymorphism found at these sites in publicly available adenovirus C sequences.

Genome site	Replicates mutated	Mutation	Sequence context	Coding	Natural polymorphism
1165	1, 2, 3	Insertion	TGGTGTGGTAATTTTTTTTTT	No	Insertions, deletions^1^
1216	2, 3	Insertion	TTGTATTGTGATTTTTTTAAA	No	Insertions, deletions^1^
7096	1, 2, 3	Insertion	TATCCTGTCCCTTTTTTTTCC	E2B	None
8617	1, 2, 3	Insertion	CCCCGGAGGTAGGGGGGGCTC	E2B	None
9417	1, 2, 3	T→G	CTGGCGGCGGTGGGGGAGGGG	E2B	None^2^
11,222	2, 3	A→C	CGGGCCCGGCACTACCTGGAC	L1	None
14,073	1, 2, 3	Insertion	GAGAATGTTTTAAAAAAAAAA	L1/No	Insertions, deletions^3^
16,602	1, 2	Insertion	GAGATCTATGGCCCCCCGAAG	L2	None
34,336	1, 2, 3	Insertion/deletion	GAAGAACCATGTTTTTTTTTT	No	Insertions, deletions^1^
35,122	2, 3	Insertion	AATAAAATAACAAAAAAACAT	No	Insertions, deletions, C/T^1^
35,215	1, 2	Insertion	GGCGTGACCGTAAAAAAACTG	E4	None

^1^ Sequence variants are shown in **S2 Fig**.

^2^C and A variants present in adenovirus B and in simian adenovirus.

**Fig 3:**
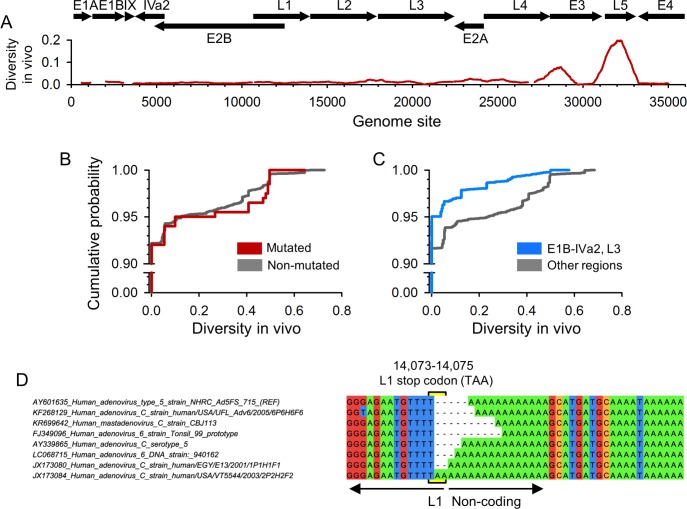
Relationship between mutation rate and the in vivo sequence diversity. **A**. Nucleotide diversity along the adenovirus genome. The protein-coding regions of different transcription units are indicated on top with arrows showing their orientation. The red line shows Nei and Li´s nucleotide diversity averaged over a 1 kpb sliding window, using alignments of adenovirus C sequences retrieved from GenBank. Some small discontinuities appear because the analysis was done of a per-gene basis to facilitate sequence alignment. **B**. Cumulative probability of nucleotide diversity values obtained from GenBank sequences, comparing sites that showed mutations in our experimental system (red) versus non-mutated sites (grey). Notice that the y-axis is broken and that most sites showed no diversity. **C**. Same figure for the two low-mutation regions (defined in **Fig 2B)** versus the rest of the viral genome. **D.** Sequence variants found around genome site 14,073 in GenBank sequences. Accession numbers are included in sequence names. We retrieved 47 adenovirus C sequences in total, but only one example per variant is shown for clarity. Similar figures for other recurrently mutated sites (see **Table 2**) are provided in **S2 Fig**.

^3^Only in the non-coding region. The L1 region contained no insertions/deletions. See **Fig 3D**.

In contrast, other similar motifs such as a poly-T 11-mer at genome positions 34,337–34,347 and a poly-T 10-mer at genome positions 1161–1170 did not show a similarly marked decrease in sequencing coverage.

### Relationship between mutation rate and sequence diversity in vivo

Under a null model in which the mutation rate shows no appreciable variation along the HAdv5 genome, mutated sites should represent a random sample of genome sites, hence, should not show particularly elevated diversity or evolvability. To test this, we downloaded from GenBank 35–52 adenovirus C sequences for each gene (except the L3 hexon gene, for which only 15 well-aligning sequences could be retrieved; **S1 Dataset**) and calculated Li and Nei´s nucleotide diversity per site, defined as the probability that pairs of sequences differ at that particular site (**Fig 3A**). Of the 29,858 genome sites examined, 2463 (8.2%) were polymorphic. Overall, the distribution of diversity values was similar for genome sites showing mutations in our experimental system and for those showing no mutations (**Fig 3B**), supporting no major effects of mutation rate variation on in vivo diversity of HAdv5. However, when we focused on the low-mutating regions L3 and E1B-IVa2, the fraction of polymorphic sites in database sequences dropped to 5.2%, versus 9.1% outside these regions (Fisher test: *P* < 0.001; **Fig 3C**), suggesting that reduced mutation rate limits in vivo diversity in these regions. We also tested whether the 11 sites showing recurrent mutations in our experimental design exhibited the same mutations in database sequences. If these sites were true mutational hotspots, database sequences should tend to show variation at these sites too. In contrast, if these were DS artefacts, most should not be polymorphic or, alternatively, they may systematically show sequence changes similar to those detected by DS if low-complexity sequence contexts also led to errors in database sequences. Interestingly, recurrently mutated sites that mapped to non-coding regions showed variation in GenBank sequences, whereas those mapping to coding regions did not (**Table 2; Fig 3D; S2 Fig**). This strongly suggests that most of the proposed mutational hotspots are real and that, in patient-derived sequences, purifying selection has removed these mutations from coding regions, but not from non-coding regions.

## Discussion

**Fig 4:**
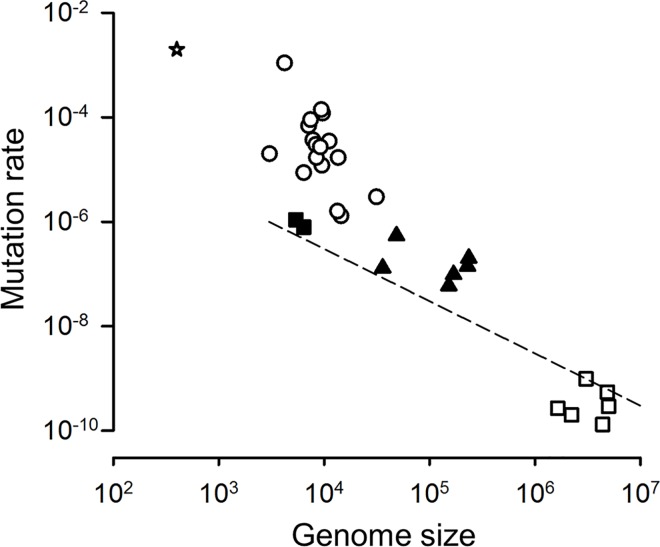
Correlation between genome sizes and mutation rates in viruses. Published mutation rate estimates are shown for a viroid (star), RNA viruses including retroviruses (white circles), single-stranded DNA viruses (black squares; from left to right: bacteriophage ØX174 and bacteriophage m13), double-stranded DNA viruses (black triangles; from left to right: HAdv5 from this study, bacteriophage λ, herpes simplex virus, bacteriophage T2, murine cytomegalovirus, and human cytomegalovirus). See text for DNA virus mutation rate references. Details on mutation rate data can be found in a recent review [26]. Bacterial and viroid mutation rates were taken from a previous review [7]. The dashed line corresponds to a mutation rate of 0.003 per site.

Per-base mutation rates correlate negatively with genome sizes over a broad range of DNA microorganisms including viruses, bacteria, and unicellular eukaryotes [7,24,25]. As a result, the genomic mutation rate varies weakly, and a quasi-constant rate of approximately 0.003 mutations per genome per round of copying was suggested [24]. For HAdv5, our calculated mutation rate per cell infection cycle is 1.3 × 10^−7^ or, equivalently, 0.0046 per 35.9 kbp genome, in good agreement with the suggested rule. In recent work with human cytomegalovirus, *de novo* mutations were identified in longitudinal patient samples and, using the estimated duration of the cell infection cycle for this virus *in vivo*, the calculated mutation rate was 2.0 × 10^−7^ [11]. Previous work with murine cytomegalovirus gave a very similar estimate of 1.4 × 10^−7^, although this mutation rate was measured per day instead of per cell infection cycle [8]. In herpes simplex virus, the mutation rate was estimated by scoring null mutations in the *tk* gene using ganciclovir [10]. This yielded an estimated rate of 5.9 × 10^−8^ per cell infection cycle [7]. Therefore, mutation rates for different human DNA viruses measured by widely different methods vary within approximately twofold around 10^−7^ mutations per base per cell infection cycle. Genome sizes range from 35.9 kbp for HAdV to 150 kpb for herpes simplex virus and 230–236 kbp for cytomegaloviruses. As a result, genomic mutation rates vary by approximately an order of magnitude, and are substantially lower than the 0.003 expected value for the largest DNA viruses (**Fig 4**).

This cast some doubts on the proposed relationship between genome sizes and mutation rates, at least for viruses. However, estimates for cytomegaloviruses and herpes virus suffer from limitations imposed by scoring only a few, potentially unrepresentative, genome sites, and from biases associated to selection, and hence should be taken with caution, making it premature to draw conclusions. We suggest, though, that factors other than genome size *per se* determine DNA virus mutation rates, such as whether the virus encodes its own polymerase or whether the viral genomic DNA is single-stranded or double-stranded. For instance, viruses encoding their own polymerases should have greater capacity to optimize mutation rates in response to selective pressures such as mutational load, the costs of replication fidelity, or adaptation to novel environments, compared to viruses that use host-encoded polymerases. Also, single-stranded DNA should be more prone to spontaneous damage and host-mediated editing than double-stranded DNA. Future work should help clarify the molecular mechanisms and evolutionary processes underlying mutation rate variation across viruses.

Whereas from an evolutionary standpoint mutation rates per cell infection cycle are meaningful because the cell infection cycle is the equivalent of a viral generation, from a biochemical perspective use of per round of copying estimates better reflects the actual fidelity of replication. If approximately 10^4^ HAdv5 infectious units are produced per cell, there should be at least log_2_ (10^4^) = 13.3 rounds of semi-conservative replication per cell, giving a rate of 1.3 × 10^−7^/ 13.3 = 9.8 × 10^−9^ per-round-of copying. Notice that this estimate is robust to gross uncertainties in burst size measurements. If, for instance, we assume only 10^3^ HAdv5 infectious units per cell, the estimated mutation rate per round of copying would change only weakly, i.e. 1.3 × 10^−7^/ log_2_ (10^3^) = 13.0 × 10^−9^. The mutation rate of normal human cells is on the order of 10^−9^ per round of copying [25]. In contrast, typical mutation rates for tumoral cells are at least 100-fold higher [27], with recent estimations for various types of cancers ranging from 10^−7^ to 10^−6^ [28]. Here, we used human cervix tumor HeLa cells for HAdv5 growth, suggesting that the HAdv5 mutation rate per round of copying was similar or even lower than that of the host cell. Whether adenoviruses use cellular post-replicative repair is unclear and the fact that tumoral cells as those used here typically show aberrant repair pathways precludes us from addressing this question here. Use of normal cells for HAdv5 growth may help clarify this point in future studies. It is well-established that most DNA viruses cause genome instability and interact with repair and DNA damage response (DDR) pathways [29,30]. For instance, the adenovirus E4orf6 protein recruits an ubiquitin ligase and promotes the proteasomal degradation of TOPBP1, an activator of DDR via ATR [31], and defects in the adenoviral E4 gene lead to the formation of concatemers of viral genomes with heterogeneous junctions [32]. However, it remains unclear whether activation or suppression of the DDR determines DNA virus mutation rates.

Although two 5 kpb regions with an over two-fold reduction in mutation rate were identified, we found no dramatic differences in mutation rate across adenovirus transcription units. This contrasts with the ability of some organisms to critically increase the mutation rate of some *loci*. Targeted hyper-mutation has been described in the immunoglobulin genes of B lymphocytes [33], contingency *loci* encoding surface proteins in some bacteria [34], and genes encoding tail fiber proteins in some DNA bacteriophages [35]. The involved mechanisms include DNA editing, polymerase slippage in DNA tandem repeats, and error-prone reverse transcription. Adenovirus genetic diversity shows ample across-gene variation and is highest in genes involved in virus-host interactions [16,17]. In principle, adenoviruses should also benefit from targeting mutations to these specific *loci*, but we found no such targeting in HAdv5. Potential hotspots were restricted to low-complexity sequence contexts and did not span entire genes but only specific nucleotide sites. Interestingly, one such possible hotspot, the poly-A motif in the 3´end of gene L1, has also been identified as a recombination hotspot and is located between a relatively conserved genome region (3´of the hotspot) and a more variable region (5´of the hotspot) [17]. It can be speculated that frequent replication errors in this low-complexity region may recruit proteins of the post-replicative system, which would increase the likelihood of recombination events. Finally, we also found that GpC dinucleotides were 2.7 times more prone to mutation than G or C bases alone. In vertebrates, cytosines in CpGs are frequently methylated and their spontaneous deamination produces thymidines, leading to high rates of C→T substitutions [36] but, in contrast, there is little evidence for methylation or elevated mutation rates at GpC motifs [37]. Furthermore, adenovirus DNA is poorly methylated [38,39] and hence this mutational pathway should be infrequent. As such, the potential mechanisms leading to increased mutation rate at GpC motifs in HAdv5 remain to be investigated.

## Methods

### Virus and cells

HAdv5 was a generous gift from Dr. Ramón Alemany (Bellvitge Biomedical Research Institute) and was propagated in HeLa cells from ATCC. Cells were free of mycoplasma as determined by a PCR test. Cells were cultured at 37°C and 5% CO_2_ in Dulbeco’s Eagle’s medium (DMEM) supplemented with 10% FBS and antibiotics.

### Plaque assay

HeLa H1 cells at 70–80% of confluency were used for plaque assays in 6-well plates. After viral adsorption, cells were washed with PBS and incubated for 4–5 days at 37°C with 5% CO_2_ in a semi-solid medium containing DMEM supplemented with 1% FBS, 1% penicillin-streptomycin, and 0.8% noble agar overlaid with a nutrient medium layer of DMEM supplemented with 1% FBS, 1% penicillin-streptomycin, glucose and GOP supplement. Cell monolayers were fixed with 4% formaldehyde, and stained with 2% crystal violet. Viral titers were expressed as plaque forming units (PFU)/mL.

### HAdv5 DNA purification

Viral infections were done in DMEM supplemented with 5–10% FBS and 1% penicillin-streptomycin until cytopathic effect was observed. The virus was first subjected to three transfers at endpoint dilutions in 96-well plates (10 days per transfer). The last transfer was then further amplified in a 10-cm plate (3 days) and the supernatant was used to inoculate five 10-cm plates of confluent HeLa cells at an MOI of 5 PFU/cell (3 days). Cells were harvested by centrifugation and washed with PBS. A suspension of approximately 10^7^ cells was incubated 30 min on ice with 1 mL cell lysis buffer (50 mM Tris-HCl pH 8.5, 150 mM NaCl and 1% Triton X-100). Cellular debris were removed by centrifugation at 14,000 rpm for 10 min at 4°C, and the supernatant was treated with 10 mg/mL RNAse A for 1 h at 37°C. Then, viral lysis buffer (10% SDS, 0.5M EDTA and 10 mg/mL proteinase K) was added and samples were incubated for 1 h at 56°C. Viral DNA was extracted with phenol/ chloroform and resuspended in ddH_2_0 for direct DS sequencing. As a sequencing control, pUC18 DNA was purified using the NucleoSpin Plasmid Kit (Macherey-Nagel, Germany). All DNA samples were quantitated using the Qubit dsDNA BR Assay kit (Life Technologies, USA) prior to sequencing.

### Duplex Sequencing

The increased accuracy of DS is based on ligation of sequencing adapters containing random yet complementary double-stranded nucleotide sequences [19]. These molecular tags allow tracing each strand of the original double-stranded template and removal of mutational artefacts that appear in only one of the two strands. DS adapters were constructed by annealing of two oligonucleotides, one of which contained a 12-nt single-stranded randomized sequence tag. Annealed primers were extended using the Klenow fragment, digested to obtain cohesive ends, and used as the final DS adapters for library preparation as previously described [13]. The purified HAdv5 DNA was fragmented using a Covaris sonicator and size selection was performed with Ampure X beads. Subsequent steps including sequencing library construction were exactly performed as detailed in previous work [13]. Samples were run on a NextSeq machine (Illumina) with 150 bp reads. FastQ files were processed with the DS software pipeline (https://github.com/loeblab/Duplex-Sequencing) using BWA 0.6.2, Samtools 0.1.19, Picard-tools 1.130 and GATK 3.3–0, and GenBank accession AY601635 as reference sequence. The computational workflow relies into three major steps: tag parsing and initial alignment, single stranded consensus sequence (SSCS) assembly, and duplex consensus sequence (DCS) assembly. Finally, the processed DCS data were realigned to the reference genome to analyze each genomic position and count mutations. Default parameters were used except for family size, which was reduced from 3 to 2 to increase the number of reads. Analysis of HAdv5 and puC18 sequencing outputs at family size 2 and 3 yielded nearly identical mutation frequencies, indicating that the reduced family size did not increase sequencing error appreciably (**S2 Table**. Accession AY601635 was used to extract HAdv5 gene coordinates, and deduced changes in protein sequences were obtained with a homemade R script. The actual sequence of the virus used in the experiments, as obtained from the DS consensus, was identical to AY601635 except for two single-base substitutions: a T→C change at position 18,813, and a G→C change at position 30,598. The DCS-final.bam.pileup text file was used to visualize insertions/deletions. The DS output is available from the NCBI SRA database (www.ncbi.nlm.nih.gov/sra; accession SRP091328).

### qPCRs

We set out to check mutations at the following genome sites: 191 (G→A), 7295 (A→G), 9417 (T→G), and 33,565 (A→G). For each, we designed three primers for SYBR green-based qPCR: one located approximately 200 bp upstream of the mutated site and two for which the 3´base matched exactly the mutated site, one containing the mutant base and another containing the wild-type base (WT, see **S3 Table** for primer information). We performed two separate qPCR reactions under the same conditions, using the mutant or wild-type primer, and a modified Taq DNA pol as provided in Agilent´s Brilliant III Ultra-Fast SYBR Green qPCR Master Mix. Since pairing of the 3´base is critical for DNA extension and Taq DNA pol has no 3´exonuclease activity, each of these two alternate qPCRs should amplify selectively the mutant or wild-type DNA. All reactions were carried using 0.3 ng of the purified HAdv5 DNA and 200 nM of each primer. Preliminary gradient PCRs were carried out using WT primers to set the annealing temperature as high as possible (67°C, 69°C, 78°C, and 68.5°C for each of the sites, respectively). The thermal qPCR profile was as follows: an initial denaturation step (95°C 3 min), 40 cycles of amplification (95°C 5 s, annealing temperature 10 s, 72°C 10 s), and a final melting cycle (95°C 30 s, 65°C 30 s, 95°C 30 s). Data analysis and Ct estimation were carried out using the AriaMx software provided by the manufacturer. Each qPCR was performed in triplicate.

### Burst size estimation

To estimate the number of PFUs produced per infected cell (burst size), HeLa cells were infected with HAdv5 at an MOI of 10 PFU/cell in a 24-well plate. The supernatant was collected at 3 and 7 days post inoculation and titrated by the plaque assay. The burst size was calculated as the number of PFUs produced per culture at day 3 divided by the number of cells, giving 9.86 × 10^3^ PFU/cell. Titers at day 7 were slightly higher, yielding a burst size of 2.60 × 10^4^ PFU/cell. Since each well of a 96-well plate contained approximately 3 × 10^4^ cells, we estimate that roughly two infectious cycles were required to fully infect a well initially containing a single PFU.

### Analysis of GenBank sequences

To infer Nei and Li´s nucleotide diversity, sequence AY601635 was used as query to carry out Blastn analyses on a per-gene basis, and all hits corresponding to human adenovirus C sequences were retrieved and aligned using the Muscle algorithm implemented in Mega 7 (www.megasoftware.net). The analysis was done gene by gene to facilitate alignment. For E3, since there are abundant alternative coding regions, we arbitrarily defined our query as a region spanning AY601635 genome positions 27,500 to 31,000. Similarly, our query for the E4 regions encompassed AY601635 genome positions 32,500 to 35,119. A, T, G, C base frequencies (*f*) at each site were obtained using a custom script and Nei and Li´s diversity was calculated as Ⅱ = 1 –*f*_A_^2^ –*f*_T_^2^ –*f*_G_^2^ –*f*_C_^2^. To analyze sequence polymorphism in sites showing recurrent mutations in our system, an approximately 300 pb region flanking the site of interest was used as query for a Blastn. Human adenovirus C sequences were retrieved and visually inspected to assess polymorphism.

## Supporting Information

S1 Table List of all mutations detected by DS in each of the three biological replicates, including insertions and deletions (Excel format). The genome site in reference sequence AY601635, mutant base, sequencing coverage, number of mutation-containing reads, transcription unit, encoded protein (gene), amino acid site, mutated codons, and amino acid substitution, if any, are provided.
(XLSX)

S2 Table Effect of minimum family size on observed mutation frequencies. The mutation count, number of bases read, and mutation frequency is shown for each of the biological replicates (R1, R2, R3) and the control plasmid for minimum family size 1, 2 (value used here), and 3 (default value).
(XLSX)

S3 Table List of primers used for qPCR. (XLSX)

S1 Fig Comparison between mutation detection by qPCR and DS. Based on DS results, we selected the following sites for qPCR analysis: 191 (G→A), 7295 (A→G), 9417 (T→G), and 33,565 (A→G). In these qPCRs, one of the two primers contained the mutation in its 3´end (mutant), whereas in parallel qPCRs we used primers with the non-mutated sequence (WT). For each of these four mutations tested, we analyzed the three HAdv5 DNAs. According to DS, mutations should be found in six of the 12 total qPCRs (1, 1, 3, and 1 for the four listed mutations, respectively; see **S1 Table**). The graph shows the ΔCt value (Ct with WT primer–Ct with mutant primer) for the 12 (6 + 6) different qPCRs performed. Each data point represents the average of three replicate qPCR assays. The error bar indicates the standard error of the mean for the six plotted data points. We found that ΔCt values were less negative in qPCRs corresponding to DS-detected mutations than in those corresponding to mutations not detected by DS (Mann-Whitney test: *P* = 0.041). However, one of the DS-detected mutations showed a highly negative ΔCt value (outlier indicated with an arrow), suggesting that the mutation was a DS artefact and was not truly present in the HAdv5 template. After removal of this outlier, differences between the two groups became more highly significant (*P* = 0.004).
(TIF)

S2 Fig GenBank sequence variants in sites showing recurrent Duplex Sequencing mutations. As in **Fig 3D**, multiple sequences were retrieved, but only one example of each variant is shown for clarity. Accession numbers are included in sequence names, and the AY601635 site is indicated on top. No alignments are shown for sites showing recurrent DS mutations but no diversity in GenBank sequences.
(TIF)

S1 Dataset List of GenBank sequences (alignment number, region/gene, sequence names, accessions) used for the analysis of genetic diversity. (XLSX)
