# Supplementary figures and images for: Genome-Wide Estimation of the Spontaneous Mutation Rate of Human Adenovirus 5 by High-Fidelity Deep Sequencing
Source: PLoS Pathog. 2016 Nov 8;12(11):e1006013. doi: 10.1371/journal.ppat.1006013 (PMC5100877; doi:10.1371/journal.ppat.1006013)

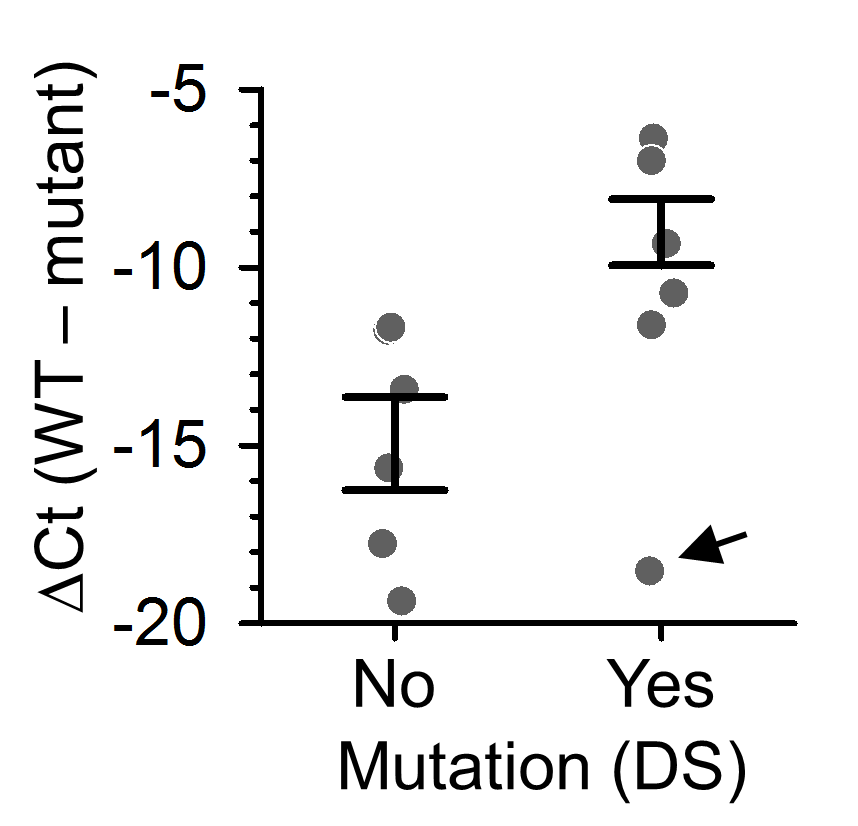

Supplement: S1 Fig — Based on DS results, we selected the following sites for qPCR analysis: 191 (G→A), 7295 (A→G), 9417 (T→G), and 33,565 (A→G). In these qPCRs, one of the two primers contained the mutation in its 3´end (mutant), whereas in parallel qPCRs we used primers with the non-mutated sequence (WT). For each of these four mutations tested, we analyzed the three HAdv5 DNAs. According to DS, mutations should be found in six of the 12 total qPCRs (1, 1, 3, and 1 for the four listed mutations, respectively; see S1 Table). The graph shows the ΔCt value (Ct with WT primer–Ct with mutant primer) for the 12 (6 + 6) different qPCRs performed. Each data point represents the average of three replicate qPCR assays. The error bar indicates the standard error of the mean for the six plotted data points. We found that ΔCt values were less negative in qPCRs corresponding to DS-detected mutations than in those corresponding to mutations not detected by DS (Mann-Whitney test: P = 0.041). However, one of the DS-detected mutations showed a highly negative ΔCt value (outlier indicated with an arrow), suggesting that the mutation was a DS artefact and was not truly present in the HAdv5 template. After removal of this outlier, differences between the two groups became more highly significant (P = 0.004). (TIF) [file ppat.1006013.s004.tif]

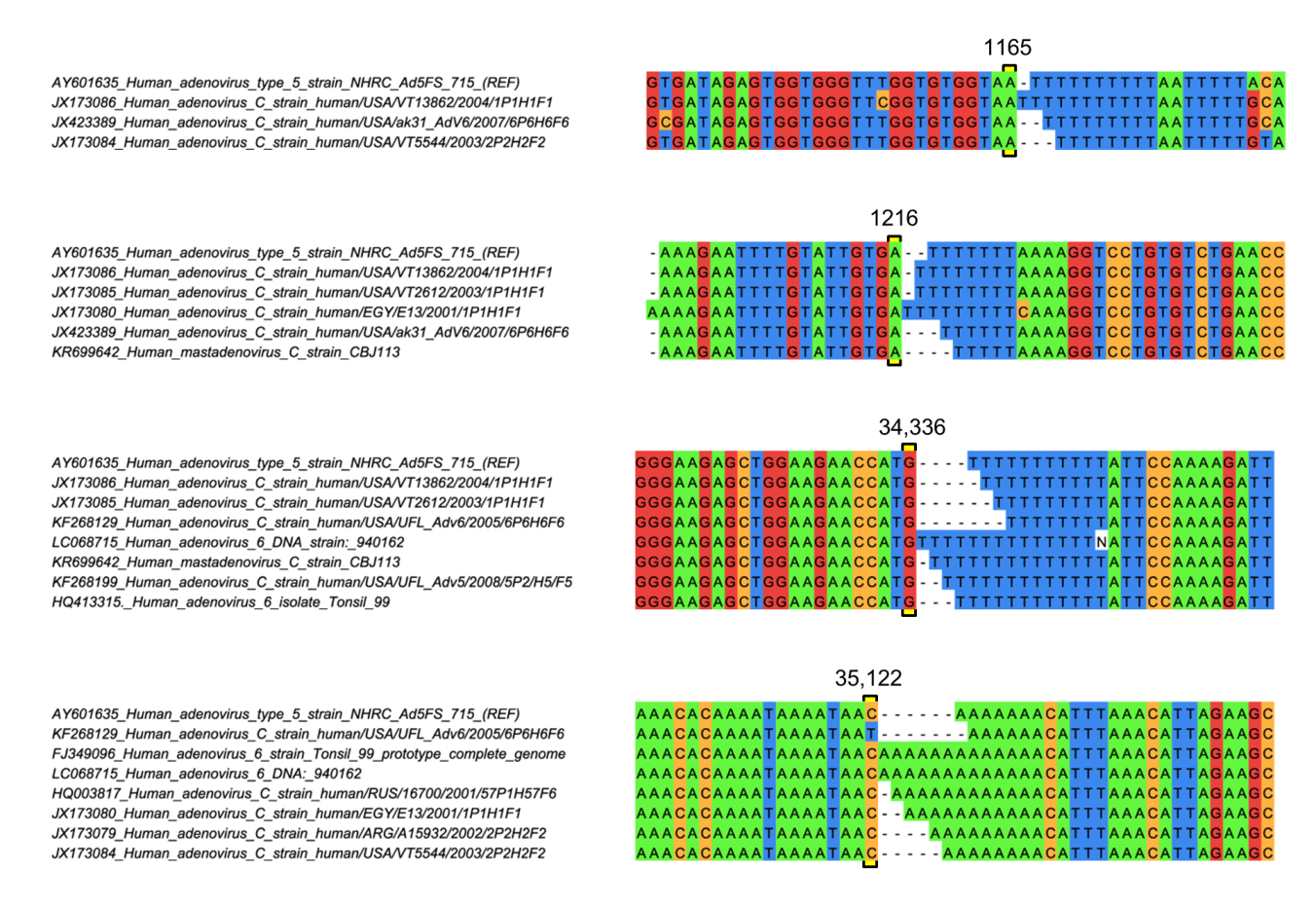

Supplement: S2 Fig — As in Fig 3D, multiple sequences were retrieved, but only one example of each variant is shown for clarity. Accession numbers are included in sequence names, and the AY601635 site is indicated on top. No alignments are shown for sites showing recurrent DS mutations but no diversity in GenBank sequences. (TIF) [file ppat.1006013.s005.tif]
